# Relative quantification of mRNA: comparison of methods currently used for real-time PCR data analysis

**DOI:** 10.1186/1471-2199-8-113

**Published:** 2007-12-20

**Authors:** Štefan Čikoš, Alexandra Bukovská, Juraj Koppel

**Affiliations:** 1 Institute of Animal Physiology, Slovak Academy of Sciences, Šoltésovej 4, 04001 Košice, Slovakia

## Abstract

**Background:**

Fluorescent data obtained from real-time PCR must be processed by some method of data analysis to obtain the relative quantity of target mRNA. The method chosen for data analysis can strongly influence results of the quantification.

**Results:**

To compare the performance of six techniques which are currently used for analysing fluorescent data in real-time PCR relative quantification, we quantified four cytokine transcripts (IL-1β, IL-6 TNF-α, and GM-CSF) in an *in vivo *model of colonic inflammation. Accuracy of the methods was tested by quantification on samples with known relative amounts of target mRNAs. Reproducibility of the methods was estimated by the determination of the intra-assay and inter-assay variability. Cytokine expression normalized to the expression of three reference genes (ACTB, HPRT, SDHA) was then determined using the six methods for data analysis. The best results were obtained with the relative standard curve method, comparative Ct method and with DART-PCR, LinRegPCR and Liu & Saint exponential methods when average amplification efficiency was used. The use of individual amplification efficiencies in DART-PCR, LinRegPCR and Liu & Saint exponential methods significantly impaired the results. The sigmoid curve-fitting (SCF) method produced medium performance; the results indicate that the use of appropriate type of fluorescence data and in some instances manual selection of the number of amplification cycles included in the analysis is necessary when the SCF method is applied. We also compared amplification efficiencies (E) and found that although the E values determined by different methods of analysis were not identical, all the methods were capable to identify two genes whose E values significantly differed from other genes.

**Conclusion:**

Our results show that all the tested methods can provide quantitative values reflecting the amounts of measured mRNA in samples, but they differ in their accuracy and reproducibility. Selection of the appropriate method can also depend on the design of a particular experiment. The advantages and disadvantages of the methods in different applications are discussed.

## Background

Reverse transcription (RT) followed by polymerase chain reaction (PCR) is at present the most sensitive method for the detection of specific RNA molecules. Quantification of nucleic acids using the PCR has been significantly simplified by the development of the real-time PCR technique, where the fluorescent signal reflecting the PCR product accumulation is detected in every amplification cycle. In biological applications examining gene expression, it is mostly not necessary to know the absolute amount of the measured mRNA (number of molecules in a sample). Relative mRNA quantification is an approach determining the amount of target mRNA in samples relative each to other. To compensate for differences in the RT-PCR input quality and quantity, the target mRNA amount in each sample is normalized to one or more internal controls. Selection of an optimal normalization strategy has been widely discussed [1,2] and is out of the scope of the present study.

Fluorescent data obtained from real-time PCR must be processed by some method of data analysis to obtain the relative quantity of target mRNA. There are several techniques used for real-time PCR data analysis and adequate attention should be paid to the selection of the appropriate method. Skern *et al.*[3] demonstrated that quantification results can vary dramatically depending on the method chosen for data analysis, and different analytical approaches may even lead to opposing biological conclusions. Methods for analysis of fluorescent real-time PCR data used in relative mRNA quantification can be classified in various ways, depending on the criteria applied. All methods determine the RT-PCR template quantity (designated "R_0_" throughout the present study) from the accumulation of the PCR product during the amplification process. Most techniques utilize exclusively the exponential phase of PCR to determine the amplification efficiency (designated "E" throughout the present study) and the R_0 _value [4,5]. The methods can be based on the determination of a "crossing point" between the PCR product fluorescence and a chosen benchmark. The benchmark is a point in the amplification curve (a graph of PCR product fluorescence versus amplification cycle number) that represents the same amounts of PCR product in every amplification. The number of amplification cycles needed to reach the benchmark is usually denoted as CP [6]. The most commonly used form of the benchmark is the threshold fluorescence (which can be set manually by the user or automatically by the software of a real-time PCR instrument), and the number of amplification cycles needed for reaching the threshold fluorescence is usually denoted as "Ct". The basic principle of the "threshold-based" methods is the same – the lower the RT-PCR template amount, the more amplification cycles are needed to reach the threshold fluorescence. Apart from the "threshold-based" methodologies that currently predominate, there are methods which use linear regression analysis of the fluorescent data from the exponential phase of PCR to determine the E and/or R_0 _values [7,8]. Moreover, a method that utilizes fluorescent data from the whole course of the amplification curve has been developed [9-11].

To compare the performance of six techniques which are currently used for analyzing fluorescent data in real-time PCR relative quantification, we determined the mRNA levels of four pro-inflammatory cytokines (IL-1β, IL-6 TNF-α, and GM-CSF) in mice with trinitrobenzene sulphonic acid (TNBS) – induced colitis. TNBS-induced colitis is a widely used experimental model for studying gut inflammatory processes such as ulcerative colitis and Crohn's disease [12].

## Results

### Relative standard curves, determination of amplification efficiency

**Figure 1:**
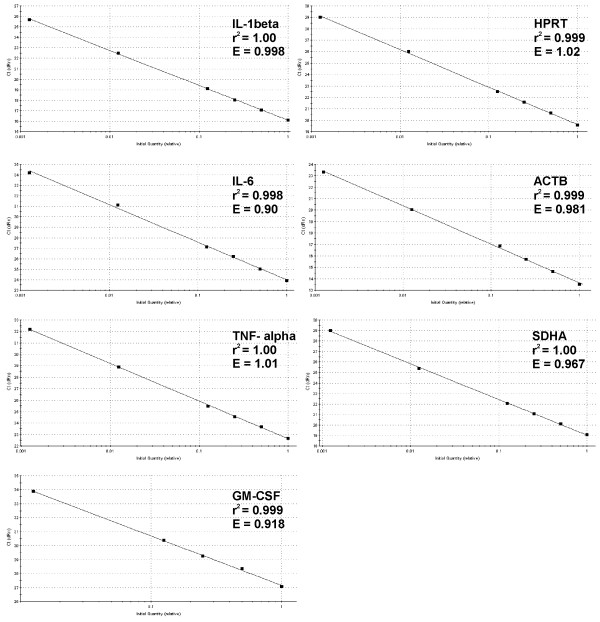
Relative standard curves. The standard curves were generated by the Mx3000P software by plotting cycles at threshold fluorescence (Ct) against the logarithmic values of standard RNA amounts. Quantities of standard RNA were expressed as dilution factors of the RNA preparation (1, 0.5, 0.25, 0.125, 0.0125, 0.00125). Correlation coefficients (square values – r^2^) and amplification efficiencies (E) are shown. SYBRGreen I fluorescences were corrected with passive reference dye (ROX) fluorescences („dRn“).

Relative standard curves of the analyzed targets are shown in Figure 1. The difference in Ct between duplicate reactions of the standard dilutions did not exceed 1% and it was in the range 0.05% – 0.75% for ACTB and in the range 0.15%–1.0% for other genes. One exception was the 800-fold dilution of IL-6 template where the difference in Ct significantly exceeded 1% (it reached 2.3%) indicating less reliable quantification at this dilution of the IL-6 template. In GM-CSF (the gene with the highest Ct values), the 800-fold template dilution did not produce a measurable fluorescent signal.

Amplification of the added luciferase mRNA showed very similar Ct values in all six dilutions of the standard RNA (arithmetical mean: 19.4, coefficient of variation: 1.28) indicating a similar efficiency of reverse transcription in all dilutions. Moreover, high correlation coefficients of the standard curves (Fig. 1) indicate that both, the efficiencies of reverse transcription and the PCR amplification efficiencies are similar in all dilutions.

Amplification efficiency (E) representative for each gene was determined from equation 7 in the relative standard curve method (Table 1). Amplification efficiency for each individual reaction was calculated using equation 10 in the DART-PCR and LinRegPCR methods; in the Liu & Saint-exp method, the amplification efficiency of each reaction was calculated from equation 11. Table 1 shows average amplification efficiencies determined for each gene using the DART-PCR, LinRegPCR and Liu&Saint-exp methods. On the whole, E values determined with the LinRegPCR and Liu &Saint-exp methods were rather lower and the ones determined with the DART-PCR method were rather higher than the E values determined from relative standard curves. In genes with low-(ACTB, IL-1β) and medium-(SDHA, HPRT, TNF-α) Ct values, the average amplification efficiencies determined by the four methods were close to 1.0; in genes with high-Ct values (IL-6, GM-CSF), the efficiencies were lower. However, the amplification efficiency of the gene with the highest Ct values (GM-CSF, average Ct value from dilutions 1× – 80×: 29.79) was higher than the efficiency of the other „high-Ct“ gene (IL-6, average Ct value from dilutions 1× – 80×: 26.69).

**Table 1:** Amplification efficiency determined by various methods of real-time PCR data analysis. Arithmetical means ± SD and coefficients of variation (in parentheses) are shown.

Gene/Method	Standard curve	DART-PCR	LinRegPCR	Liu&Saint-exp
IL-1β	0.998	1.033 ± 0.061 (5.95)	0.950 ± 0.074 (7.76)	0.974 ± 0.033 (3.42)
IL-6	0.900	0.912 ± 0.046 (5.05)	0.881 ± 0.075 (8.51)	0.870 ± 0.029 (3.35)
TNF-α	1.012	1.033 ± 0.024 (2.32)	0.974 ± 0.072 (7.45)	0.991 ± 0.032 (3.32)
GM-CSF	0.918	0.978 ± 0.044 (4.49)	0.932 ± 0.063 (6.75)	0.898 ± 0.013 (1.44)
ACTB	0.981	1.086 ± 0.046 (4.21)	0.999 ± 0.031 (3.10)	0.978 ± 0.044 (4.53)
SDHA	0.967	1.072 ± 0.052 (4.88)	0.980 ± 0.064 (6.55)	1.017 ± 0.044 (4.27)
HPRT	1.022	1.069 ± 0.064 (5.95)	0.978 ± 0.109 (11.1)	0.976 ± 0.048 (4.49)

### Determination of target mRNA quantity in known sample dilutions

Linear regression analysis generated by plotting values of target mRNA quantities (R_0_) against diluting factors of the total RNA preparation was performed to evaluate the accuracy how the R_0 _values determined by different methods of real-time PCR data analysis reflect the RT-PCR template dilutions. The relationship between determined R_0 _values and sample dilutions was closest in the standard curve method, comparative Ct method, DART-PCR method with average E values, Liu & Saint-exp method with average E values and the combined LinRegPCR-Ct method using average E values, where average Pearson's correlation coefficients (obtained from the coefficients of the seven genes) reached value at least 0.999 (Table 2). In the sigmoid curve-fitting method the average Pearson's correlation coefficient was 0.9953; manual selection of the cut-off cycle (see Methods) was necessary in SDHA, improving the value of the Pearson's correlation coefficient from 0.9986 to 0.9999. In the remaining three methods (LinRegPCR, DART-PCR and Liu & Saint-exp methods with individual E values) the average Pearson's correlation coefficients reached values between 0.9577 and 0.9733 (Table 2). In most cases, the Pearson's correlation coefficients in the gene with the highest Ct values (GM-CSF) were lower than in other genes.

**Table 2:** Pearson's correlation coefficients obtained from the linear regression plotting R_0 _values against diluting factors of the total RNA (RT-PCR template). The R_0 _values were obtained by transformation of fluorescence data using the following methods for real-time PCR data analysis: St cur, relative standard curve; Comp, comparative Ct; SCF, sigmoid curve-fitting; DART ind E, DART-PCR with individual E values; DART av E, DART-PCR with average E values; Liu&S ind E, Liu & Saint-exp with individual E values, Liu&S av E, Liu & Saint-exp with average E values; LinReg ind E, LinRegPCR (using individual E values); LinReg-Ct av E, LinRegPCR combined with Ct (using average E values).

Gene/Method	St cur	Comp	SCF	DART ind E	DART av E	Liu&S ind E	Liu&S av E	LinReg ind E	LinReg-Ct av E
IL-1β	0.9993	0.9996	0.9960	0.9924	0.9997	0.9697	0.9994	0.9113	0.9994
IL-6	0.9998	0.9998	0.9951	0.9745	0.9993	0.9510	0.9996	0.9391	0.9997
TNF-α	0.9996	0.9998	0.9987	0.9910	0.9989	0.9622	0.9996	0.9835	0.9996
GM-CSF	0.9980	0.9980	0.9803	0.9620	0.9985	0.9277	0.9975	0.9426	0.9980
ACTB	0.9991	0.9992	0.9973	0.9435	0.9972	0.9915	0.9998	0.9828	0.9984
SDHA	0.9998	0.9999	0.9999	0.9799	0.9999	0.9872	0.9999	0.9902	0.9999
HPRT	0.9996	0.9995	0.9997	0.9699	0.9990	0.9662	0.9994	0.9545	0.9997
Average	**0.9991**	**0.9994**	**0.9953**	**0.9733**	**0.9990**	**0.9651**	**0.9993**	**0.9577**	**0.9992**

We normalized the quantity of cytokines (IL-1β, IL-6, TNF-α, GM-CSF) with the quantity of one reference gene (ACTB, a "low-Ct" gene or HPRT, a "high-Ct" gene) or with the normalization factor to test the influence of the normalization on the accuracy of the evaluated methods. Normalization factors were determined in all dilutions of the RT-PCR template using the geNorm software. The parameter "M", calculated by the software, is a measure of the gene expression stability; the lower is the M value the higher is the gene expression stability. The parameter "V2/V3" (i.e., the third gene added to the two genes) represents pairwise variations determining the optimal number of reference genes which should be included for calculation of a reliable normalization factor [13]. Parameters of the geNorm software calculated for the three reference genes (used for the normalization factor calculation) are shown in Table 3. Values of the M parameter for ACTB, SDHA, and HPRT were similar within all methods of analysis. However, comparison of the M values between individual methods of analysis showed that the M values were 2 – 10 fold higher in the three methods utilizing individual E values (DART-PCR, Liu & Saint-exp and LinRegPCR with individual E) than in other methods. Similarly, values of the V2/3 parameter were higher in the three methods utilizing individual E values than in other methods indicating a requirement of higher amount of reference genes to calculate a reliable normalization factor.

**Table 3:** Parameters of the geNorm software for ACTB, SDHA, and HPRT. The parameters were determined from quantities (R_0 _values) of the three genes in the RT-PCR template dilutions. M – measure of the gene expression stability, V2/V3 – pairwise variations determining the optimal number of reference genes. Indicated methods for real-time PCR data analysis were used for the transformation of fluorescence data to the R_0 _values. Designation of the methods is the same as in Table 2.

Parameter/Method	St cur	Comp	SCF	DART ind E	DART av E	Liu&S ind E	Liu&S av E	LinReg ind E	LinReg-Ct av E
M for ACTB	0.159	0.161	0.315	0.598	0.200	0.544	0.213	1.772	0.276
M for SDHA	0.187	0.191	0.293	0.578	0.183	0.538	0.326	1.549	0.246
M for HPRT	0.181	0.165	0.324	0.631	0.226	0.626	0.228	1.349	0.338
V2/V3	0.057	0.060	0.097	0.190	0.071	0.195	0.107	0.564	0.109

**Figure 2:**
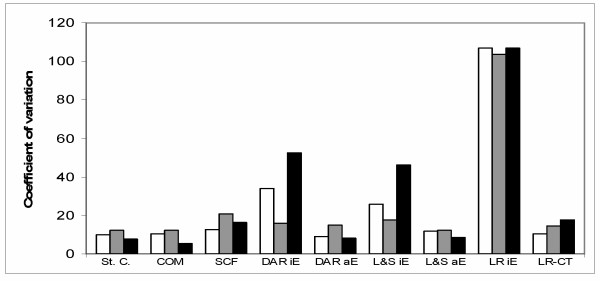
Coefficients of variation for normalized quantity of IL-1β in serial dilutions of the RT-PCR template. Quantities of IL-1β, HPRT, SDHA and ACTB mRNAs (the R_0 _values) were determined in each dilution of the RT-PCR template (six dilutions of total RNA) using all the tested methods of real-time PCR data analysis. The amount of IL-1β mRNA in each dilution was then divided by the relative amount of HPRT, ACTB, or by the normalization factor (NF, geometric mean of HPRT, SDHA and ACTB amounts) of the dilution. Arithmetical mean, standard deviation and coefficient of variation of the normalized IL-1β quantity for each type of normalization (HPRT, ACTB, NF) and each method of real-time PCR data analysis was then calculated. Coefficients of variation (CV) are shown: the first columns are CV values after normalization with the normalization factor, the second columns are CV values after normalization with ACTB, and the third columns are CV values after normalization with HPRT. Methods for real-time PCR data analysis: St. C., relative standard curve; COM, comparative Ct; SCF, sigmoid curve-fitting; DAR iE, DART-PCR with individual E values; DAR aE, DART-PCR with average E values; L&S iE, Liu & Saint-exp with individual E values, L&S aE, Liu & Saint-exp with average E values; LR iE, LinRegPCR (using individual E values); LR-Ct, LinRegPCR combined with Ct (using average E values)

Since the determined quantity of cytokines and reference genes should reflect the template dilutions by the same manner, the same normalized quantity of cytokines should be determined in each dilution. Variability of the normalized quantity between the known template dilutions can then be used as a measure of the quantification accuracy – the lower the variability between dilutions the higher the accuracy of quantification. Figure 2 shows coefficients of variation (CV) for normalized quantity of IL-1β in six RT-PCR template dilutions (similar results were obtained for IL-6, TNF-α, and GM-CSF; data not shown). In most methods, similar CV values were found after normalization to the normalization factor, to the „low Ct“-gene or to the „high Ct“-gene (which is caused by similar expression stability of the three reference genes as shown in Table 3); differences detected by the three methods utilizing individual E values were not consistent nor among the three methods neither among the four measured cytokines. On the other hand, comparison of CV values between individual methods of analysis showed markedly higher coefficients of variation in the three methods utilizing individual E values (DART-PCR, Liu & Saint-exp and LinRegPCR with individual E) than in other methods (Fig. 2).

### Intra-assay and inter-assay variability

Reproducibility of the tested methods for real-time PCR data analysis was evaluated by performing experiments where the intra- and inter-assay variability was measured. Two genes were selected for the measurements – IL-1β, representing a target with low Ct values and IL-6, representing a target with high Ct values. Results from the experiment measuring the intra-assay variability are shown in Table 4. In both genes, the lowest coefficients of variation were obtained when the following methods of real-time PCR data analysis were used for the determination of target mRNA quantity (R_0_): standard curve method, comparative Ct method, DART-PCR method with average E values, Liu & Saint-exp method with average E values and the combined LinRegPCR-Ct method (using average E values). Using the sigmoidal curve-fitting method, the coefficients of variation were markedly higher. The highest coefficients of variation were obtained using the LinRegPCR method, DART-PCR method and Liu & Saint-exp method with individual E values. Similar results (except of the SCF method) were obtained in the experiment measuring the inter-assay variability (Table 5). Coefficients of variation calculated from Ct values of 15 replicate PCR reactions in the intra-assay experiment were 0.52 for IL-1β and 0.63 for IL-6; in the inter-assay experiment, the coefficients were 1.1 for IL-1β and 1.18 for IL-6.

**Table 4:** Intra-assay variability. Coefficients of variation calculated from R_0 _values of 15 replicate PCR reactions. Indicated methods for real-time PCR data analysis were used for the transformation of fluorescence data to the R_0 _values. Designation of the methods is the same as in Table 2.

Gene/Method	St cur	Comp	SCF	DART ind E	DART av E	Liu&S ind E	Liu&S av E	LinReg ind E	LinReg-Ct av E
IL-1β	5.95	5.95	17.1	60.6	7.63	25.03	7.33	49.08	5.71
IL-6	9.94	9.94	22.3	83.6	10.2	43.7	9.28	53.5	9.73

**Table 5:** Inter-assay variability. Coefficients of variation calculated from R_0 _values of 8 PCR reactions. Indicated methods for real-time PCR data analysis were used for the transformation of fluorescence data to the R_0 _values. Designation of the methods is the same as in Table 2.

Gene/Method	St cur	Comp	SCF	DART ind E	DART av E	Liu&S ind E	Liu&S av E	LinReg ind E	LinReg-Ct av E
IL-1β	12.4	12.4	15.3	79.7	18.3	30.3	17.7	68.8	11.2
IL-6	18.6	17.1	20.5	65.5	17.7	40.2	17.5	53.8	16.2

In the standard curve method, comparative Ct method, and the three methods using average E values (DART-PCR, Liu & Saint-exp, LinRegPCR-Ct – with average E), the intra-assay variability was higher in IL-6 than in IL-1β; a similar trend was found for the inter-assay variability (except of two methods – DART-PCR and Liu & Saint-exp, which showed similar values for both genes). The three methods (DART-PCR, Liu & Saint-exp, LinRegPCR-Ct) utilizing individual E values (and showing high CV values) gave inconsistent results and in some cases, the determined intra-assay variability was even higher than the inter-assay variability. The CV values obtained in the intra- and inter-assay experiment with the SCF method were comparable, and they were higher in IL-6 than in IL-1β which is in accordance with the five well-working methods (Table 4, Table 5).

### Normalized cytokine expression in the experimental samples

**Figure 3:**
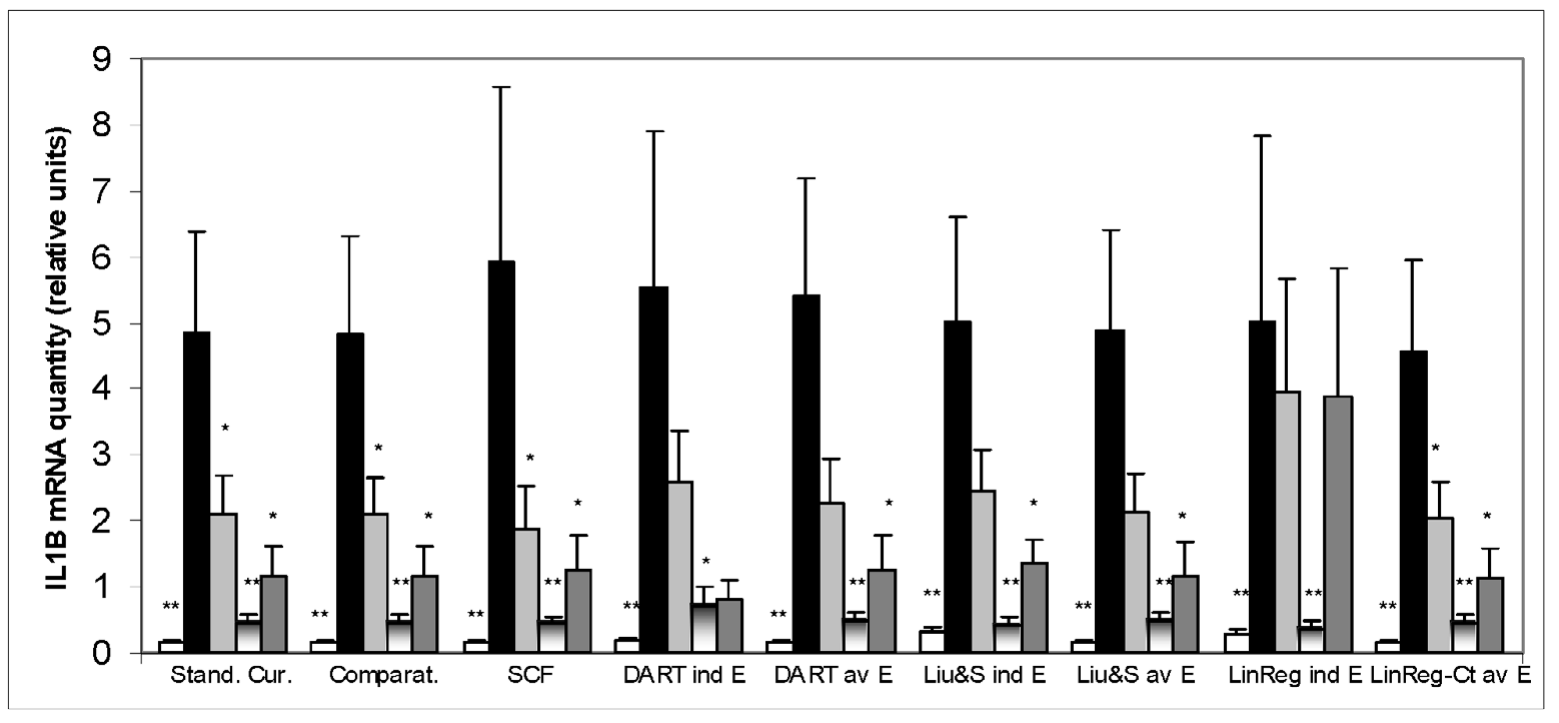
IL-1β mRNA expression. Quantities of IL-1β, HPRT, SDHA and ACTB mRNAs (the R_0 _values) were determined in each sample using all the tested methods of real-time PCR data analysis. The amount of IL-1β mRNA in each sample was then divided by the normalization factor (geometric mean of HPRT, SDHA and ACTB amounts) of the sample. Values are arithmetical means + SEM, n = 5–8. Statistical significance of the differences between the group of untreated colitic animals (Un) and other groups of animals was assessed with the Mann-Whitney test: * P ≤ 0.05, ** P ≤ 0.01. Methods for real-time PCR data analysis: Stand curv, relative standard curve; Comparat, comparative Ct; SCF, sigmoid curve-fitting; DART ind E, DART-PCR with individual E values; DART av E, DART-PCR with average E values; Liu&S ind E, Liu & Saint-exp with individual E values, Liu&S av E, Liu & Saint-exp with average E values; LinReg ind E, LinRegPCR (using individual E values); LinReg-Ct av E, LinRegPCR combined with Ct (using average E values) Designation of the animal groups: the first columns, control sham animals (Sh); the second columns, untreated colitic animals (Un); the third columns, colitic animals with the treatment A; the fourth columns, colitic animals with the treatment B; the fifth columns, colitic animals with the treatment C

**Figure 4:**
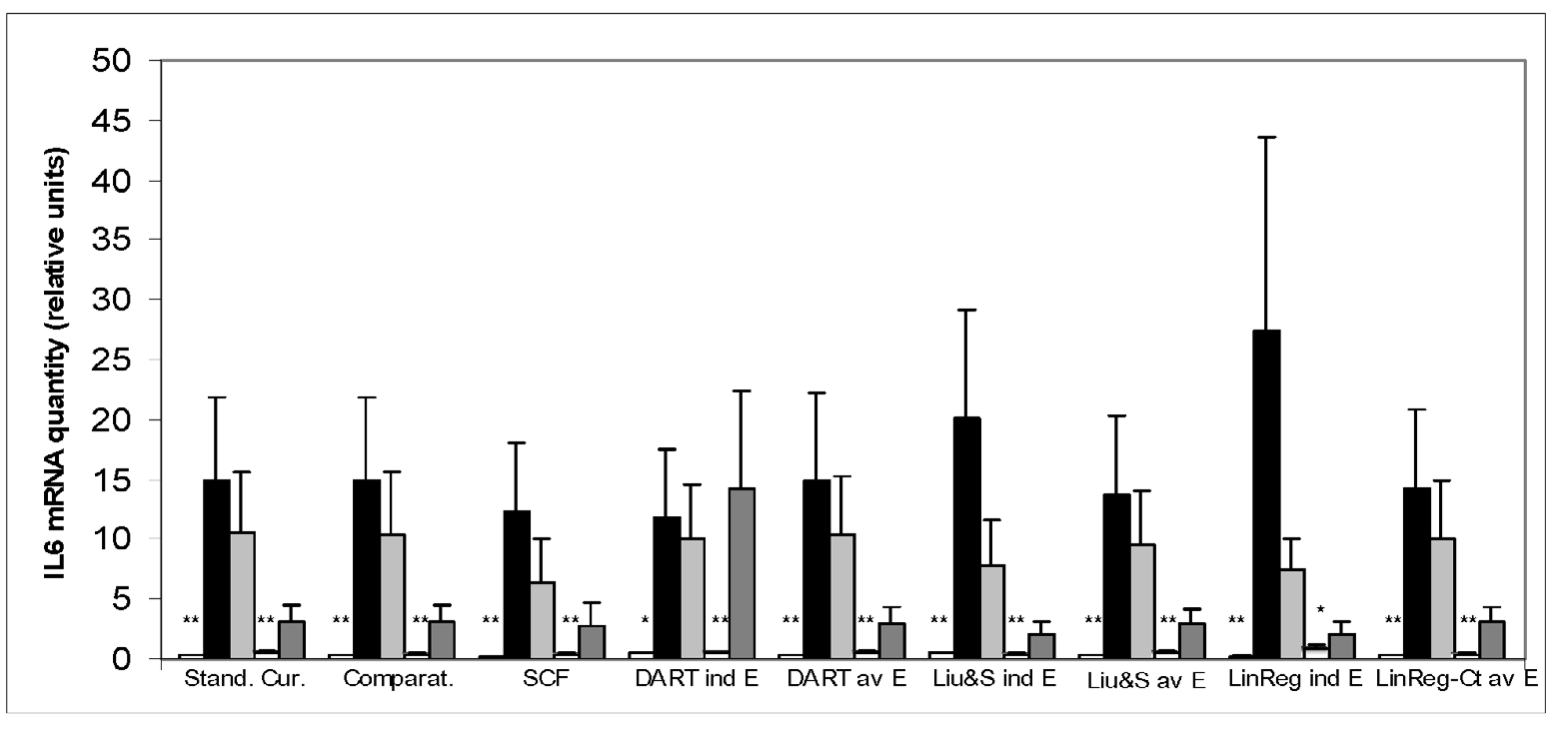
IL-6 mRNA expression. Quantities of IL-6, HPRT, SDHA and ACTB mRNAs (the R_0 _values) were determined in each sample using all the tested methods of real-time PCR data analysis. The amount of IL-6 mRNA in each sample was then divided by the normalization factor (geometric mean of HPRT, SDHA and ACTB amounts) of the sample. Values are arithmetical means + SEM, n = 5–8. Statistical significance of the differences between the group of untreated colitic animals (Un) and other groups of animals was assessed with the Mann-Whitney test: * P ≤ 0.05, ** P ≤ 0.01. Designation of the methods and animal groups is the same as in Figure 3.

Figure 3 and Figure 4 show normalized expression of IL-1β and IL-6 as determined by all the tested methods of real-time PCR data analysis in the experimental samples. Estimation of IL-1β and IL-6 expression using the Kruskal-Wallis test showed that differences between the experimental groups were highly significant (P ≤ 0.01 using the LinRegPCR method with individual E values and P ≤ 0.001 with all other methods). Estimation of the difference in IL-1β and IL-6 expression between the group Un (untreated colitic animals) and the other groups of animals (with the Mann-Whitney test) showed significantly increased expression of these cytokines in the untreated colitic animals, using all the tested methods of real-time PCR data analysis. All methods also detected a significant decrease of the two cytokines expression after treatment B (with lower level of significance using LinRegPCR or DART-PCR methods with individual E values). In the case of IL-1β, some of the methods also detected significant decrease in the cytokine expression after treatments A, or C.

In TNF-α and GM-CSF, significant differences were found only between the group of untreated colitic animals (Un) and the group of sham control animals (Sh). In TNF-α, all the tested methods of real-time PCR data analysis showed significantly higher amount of the cytokine in the group Un than in the group Sh. In GM-CSF, the difference between the group Un and Sh was detected as significant only with the standard curve method, comparative Ct method, and the three methods utilizing average E values (DART-PCR, Liu & Saint-exp, LinRegPCR-Ct – with average E); the methods which used individual E values (DART-PCR, Liu & Saint-exp, LinRegPCR-Ct – with individual E) did not detect the difference as statistically significant (data not shown).

## Discussion

To compare the methods currently used for analyzing fluorescence data in real-time PCR relative quantification, we determined mRNA levels of four pro-inflammatory cytokines in the *in vivo *model of colonic inflammation, using six different techniques. Three housekeeping genes were also quantified to serve as reference genes for the normalization of cytokine mRNA quantity. The compared methods differ principally in the mathematical function used for modeling the PCR process, in the necessity to create a dilution series of the RT-PCR template, in the necessity and means of amplification efficiency (E) determination, in the way of calculation of the target mRNA quantity (R_0_).

Most of the methods utilize fluorescence data only from the exponential phase of PCR amplification (the exponential model of PCR) and require setting a threshold fluorescence (common for all compared samples) to determine the Ct value ("threshold-based" methodologies). The relative standard curve method determines target mRNA relative quantities in samples from known relative quantities of standard RNA or cDNA. To obtain correct results, amplification efficiency in the dilutions of the standard preparation and in samples must be similar. The E value determined from the slope of the standard curve (or from a dilution series of a representative sample) can be utilized for the calculation of the target mRNA quantity (R_0_) in techniques of relative quantification, which can be denoted as "comparative Ct methods". Equation 5 can be used for the transformation of Ct values to the R_0 _values; the only parameter which differs among the compared samples is Ct. The term "comparative Ct method" is most frequently used for the " 2^-ΔΔCt ^" method introduced by Livak [14]; in this method E is assumed to be equal to 1 and the formula for the R_0 _calculation can be modified to R_0 _= 2^-Ct^. Pfaffl's model of the comparative Ct method [15] incorporates a correction for amplification efficiency differing from the optimal value 1. Most published versions of the comparative Ct method use normalization of the target gene quantity to a reference gene quantity, very often referring to one of the samples (a "calibrator" sample), and the formula for calculation of the target gene relative quantity is more complicated [14-19]. In any case, formulas used in comparative Ct methods for calculation of the normalized relative amount of target gene quantity can be derived from equation 5. Pfaffl *et al.*[20] developed the software named REST (Relative Expression Software Tool) which can perform the comparative Ct quantification in two experimental groups (with or without the E value correction) followed by a statistical test. In the present study, we used E values determined for each gene from the relative standard curves and calculated the R_0 _value for each gene in each sample using the equation 5. The cytokine genes quantity was then normalized with the factor obtained from the quantities of three reference genes.

In the method proposed by Liu and Saint [21], the amplification efficiency for each sample is determined from the amount of fluorescence and the number of cycles at two arbitrary fluorescence thresholds along the exponential phase of the PCR amplification (Eq 11). The fluorescence thresholds can be set individually for each amplification, or a threshold level common for all reactions can be set. In the first case equation 4 is used for the R_0 _calculation, and in the second case equation 4 or equation 5 can be used. We compared the quantification results obtained with this method using either individual E values (determined for each amplification) or using an average E value.

Ramakers *et al.*[7] developed a computer program entitled LinRegPCR, which determines the target mRNA quantity (*R*_0_) and amplification efficiency (E) by linear regression analysis (Eq 9, Eq 10) of fluorescence data obtained from real-time PCR. Like the above-mentioned methods, linear regression exploits only the exponential phase of PCR amplification, but the method is not "threshold-based" – no benchmark (threshold fluorescence) is needed for the calculations. We analyzed our fluorescence data with the LinRegPCR software and with a technique which combines linear regression analysis and the threshold-based methodology. The combined technique (we have designated it "LinRegPCR-Ct") utilizes E values determined by the LinRegPCR software (for calculation of an average E) and Ct values determined by the Mx3000P real-time PCR instrument. A similar strategy was applied by Karlen *et al.*[22] and Schefe *et al.*[23]. The DART-PCR program developed by Peirson *et al.*[8] provides an automated analysis of real-time PCR fluorescence data utilizing the combined approach (linear regression for E determination and threshold fluorescence for Ct determination). Similarly as for LinRegPCR, we compared the quantification results obtained with DART-PCR using individual or average E values for the R_0 _calculation.

The method in which a sigmoid mathematical model that fits the kinetics of the whole real-time PCR process is applied [9,10], represents an approach completely different from the above-mentioned techniques. The sigmoid curve-fitting (SCF) method utilizes all fluorescence data recorded during the amplification process (not only the data from the exponential phase) for determination of the R_0 _value. Moreover, the method can carry out quantification without the knowledge of amplification efficiency and without determination of Ct. Rutledge [11] found that amplification cycles within the plateau phase of PCR deviate from that predicted by sigmoid curve-fitting, and their exclusion from the curve-fitting process is necessary. He proposed the selection of a cut-off cycle beyond which further cycles are excluded from the fitting of the amplification curve. The criterion used for the selection of the cut-off cycle was based on repetitive curve-fitting in which the last cycle was sequentially excluded and the R_0 _value was calculated at each individual curve-fitting. Plotting the calculated R_0 _values against the cut-off cycle revealed a highly regular pattern in which the calculated R_0 _value decreased with subsequent cycle removal, and after reaching a minimum a small increase in the R_0 _value appeared; the minimum-calculated R_0 _value was selected as the resulting R_0_. In our work, we found that the shape of the graph of R_0 _dependency on the cut-off cycle can be influenced by fluorescence data provided by the real-time PCR system. Using background-subtracted fluorescence data from the Mx 3000P system (Stratagene), we were not able to identify the regular trend of the curve R_0 _vs cut-off cycle as described by Rutledge [11]; background-subtracted data from Opticon2 DNA Engine (MJ research Inc.) were used in Rutledge's study. On the other hand, raw fluorescence data from Mx 3000P provided a regular pattern of the R_0 _value as a function of the cut-off cycle (R_0 _decrease followed by a small increase after reaching a minimum) in most curves. In some instances the cut-off cycle with the minimal R_0 _value was determined in the region of the amplification curve which contained an insufficient amount of fluorescent data, and manual selection was necessary. Karlen *et al.*[22] testing the performance of several methods for real-time PCR data analysis found the sigmoid curve-fitting method (together with the method fitting PCR amplification to the exponential function) as the least suitable for quantitative PCR analysis. On the contrary, our results indicate that the SCF method can provide reasonable results. Similarly, Qiu *et al.*[24] obtained comparable results using the SCF method and a classic threshold-based method. The differences in the SCF method performance were probably caused by different number of amplification cycles included into the fitting process. An appropriate selection of the optimal cycle number (exclusion of late cycles) is probably the key factor for obtaining satisfactory performance of the SCF method, but the choice of a suitable criterion for determination of the "cut-off cycle" can be difficult (as discussed above).

In our study, we determined amplification efficiencies (E) of seven genes using four methods of real-time PCR data analysis, and found some differences in the determined E values. On the other hand, all the methods were capable to identify the two genes whose E values significantly differed from the others. Interestingly, the amplification efficiencies determined by the two methods which employ linear regression for the calculation (DART-PCR and LinRegPCR) were less close each to other than to efficiencies determined by the relative standard curve or Liu & Saint-exp method. This can be caused by differences in the way the two methods determine the exponential phase of amplification. Lower amplification efficiency found by all tested methods in two genes with high Ct values (IL-6 and GM-CSF) could suggest some influence of Ct value on the determination of the E value. However, the comparison of E and Ct values in the two „high-Ct genes“ indicates that a higher Ct value is not necessarily leading to obtaining of a lower E value. This finding is in accordance with results of Karlen *et al.*[22] who did not find a dependency of E value on Ct value; the authors defined the amplicon and primer sequences as the main factor influencing the efficiency of amplification.

To compare the accuracy of relative quantification conducted with application of different methods for real-time PCR data analysis, we determined quantities of seven target mRNAs in serially diluted preparation of total RNA. We found that R_0 _values determined with the relative standard curve method, comparative Ct method and with the three methods using an average E value for the calculations (DART-PCR, Liu & Saint-exp, LinRegPCR-Ct – with average E) most accurately reflected the RT-PCR template dilutions. Less effective was the SCF method, and the worst results were obtained with the three methods which used individual E values (DART-PCR, Liu & Saint-exp, LinRegPCR – with individual E). Normalization of cytokines mRNA quantity (to a single reference gene or to a normalization factor calculated from reference genes with comparable expression stability) did not influence the differences in the performance of the methods for real-time PCR analysis tested in our study. On the other hand, our results indicate that targets with high Ct values can be quantified with a lower accuracy than targets with medium and low Ct values. Reproducibility of the tested methods was estimated by determination of the intra- and inter-assay variability and showed the same result as the accuracy test. The highest reproducibility was found in the relative standard curve method, comparative Ct method and in the three methods using an average E value for calculations. The SCF method was less precise, and the worst results were obtained with the three methods which used individual E values. Our results also showed a negative effect of higher Ct values on the reproducibility of the tested methods.

Comparing results of normalized expression of IL-1β and IL-6 in the experimental samples, all the tested methods were able to detect significant changes between the control animals, untreated colitic animals, and animals undergoing treatment B. The effects of treatments A or C on the IL-1β mRNA level were detected as statistically significant only by some of the tested methods. In TNF-α and GM-CSF, all the tested methods showed higher amount of the cytokines in untreated colitic animals than in control animals, hovewer the three methods which utilized individual E values (DART-PCR, Liu & Saint-exp, LinRegPCR-Ct – with individual E) were not able to detect the difference in GM-CSF expression as statistically significant.

Application of corrections for individual sample efficiency should theoretically improve the accuracy and reproducibility of the quantification. But our results showed that, on the contrary, the use of individual E values for the R_0 _calculation impaired the quantification. Similar findings were presented in studies where linear regression was utilized for the calculation of individual amplification efficiencies [8,22,23]. We used two approaches for determination of the individual amplification efficiencies – linear regression (in DART-PCR method and LinReg PCR method which differ in the way of exponential phase determination) and the method which utilize setting of two fluorescence thresholds along the exponential phase (Eq 11). Independently on the way used for the calculation of E values, all the three methods showed the negative effect of the individual E values on the quantification which indicates that this is probably a feature connected with the limited precision of individual data and not with the mathematical approach used for E value determination. The individual E values (determined with linear regression or with the Eq 11) are derived from individual sample kinetics represented by the fluorescence values obtained in particular amplification cycles. Above mentioned results suggest that fluorescence values detected by real-time systems do not reflect the reaction kinetics with the precision which would be sufficient for reliable E value determination from the exponential phase of the individual amplification. The average E value obtained from the group of amplifications eliminates individual imprecisions enabling to find a reliable value of the amplification efficiency.

## Conclusion

Choosing the appropriate method for real-time PCR data analysis can depend on conditions in a particular application. The relative standard curve method is widely used and can provide reliable results, especially in the case when the same quality of RT-PCR template is ensured in standards and samples. Sometimes this is not possible, for instance if the quantification is performed on different tissue types, or if the amount of tissue is limited (e.g. tissue biopsies, preimplantation embryos). Similar restrictions apply also for comparative Ct methods. These methods require serial dilutions of a representative sample to determine the E value which is usually done in a validation experiment preceding a series of measurements. This approach enables to measure more samples in a PCR run (no standard curve in the run), but identical conditions for all measurements must be ensured. The other three methods tested in the present study (Liu & Saint-exp, DART-PCR, LinRegPCR) do not require serial dilutions of the RT-PCR template for E value determination. We found that these methods provide more reliable results when an average E value and not individual E values (determined for each amplification) are used for the R_0_calculation. The Liu & Saint-exp method is simple – the formula for E value calculation can be easily implemented into spreadsheet programs such as Microsoft Excel and the selection of the exponential phase of amplification is not difficult (the human eye is good enough for distinguishing a straight line). Our results with the Liu & Saint-exp quantification showed that although the criterion for selection of the exponential phase is subjective, reasonable results can be obtained if the same criterion is applied for all compared samples. The LinRegPCR and DART-PCR methods use a more complicated calculation (based on linear regression) for the E value determination. The DART-PCR software combining the linear regression analysis with threshold-based methodology enables R_0 _values to be calculated using an average E value; the LinRegPCR software do not enable the automated use of an average E value, but manual combination with a threshold-based technique is possible. The SCF method differs from all the other methods in the mathematical model used for the calculations – it is not necessary to look for the exponential phase of amplification and to determine the E value. For reliable quantification with this method, fluorescence data including at least the beginning of the plateau phase are needed, which can be a disadvantage when genes with low expression are quantified or when low sample amounts are available. In summary, our results show that all the tested methods for real-time PCR data analysis can provide quantitative values reflecting the amounts of measured mRNA in samples, but they differ in their accuracy and reproducibility. Although selection of the appropriate method can be limited by the design of a particular experiment (e.g. tissue type, gene abundance, number of experimental groups) the use of more than one analytical method is recommended for validation of results.

## Methods

### Animal experiment, sample preparation, and real-time PCR

All the fluorescent data used in this study were obtained from a previous study examining the effects of plant essential oils thyme and oregano on trinitrobenzene sulphonic acid (TNBS)-induced colitis in mice [25]. Briefly, colitis was induced in male Balb/c mice by administration of TNBS and the animals were treated with three increasing concentrations of the plant oils (treatments A, B and C; the group of untreated colitic animals was designated as Un and sham control group as Sh in the present study). Total RNA was isolated from the strips of colonic tissue with TRIzol Reagent (Invitrogen Life Technologies, Karlsruhe, Germany). The RNA preparations were then cleaned and DNase I treated with an RNeasy Micro kit (Qiagen, Hilden, Germany). Complementary DNA (cDNA) was then synthesized from the RNA (0.75 μg from each sample) using Superscript™ II Rnase H-Reverse Transcriptase (Invitrogen Life Technologies; for more details see [25])

Serial dilutions of total RNA (1× – i.e. no dilution, 2×, 4×, 8×, 80×, 800×) were prepared from the pool of colon RNA obtained by combining aliquots of samples from the colitic animals. Complementary DNA was then synthesized from each dilution as described above. To compensate for different RNA amounts in the reverse transcription reactions yeast total RNA was added in appropriate amounts to the colon RNA dilutions (all oligonucleotide primers were checked so as not to create any PCR product on the yeast cDNA template). The cDNAs then served as a PCR template for construction of relative standard curves. Amplifications performed on the standard cDNAs were also utilized for comparison of the accuracy of quantification by the tested methods for real-time PCR data analysis (determination of known sample dilutions); in the relative standard curve method two sets of total RNA dilutions (and cDNA preparations) were used – one set was utilized for the construction of standard curves and the other set served as known sample dilutions.

To test the efficiency of reverse transcription in the serial dilutions of standard RNA, identical amount (10 pg) of luciferase mRNA (Promega, Madison, WI) was added into the six RNA dilutios (1×, 2×, 4×, 8×, 80×, 800×) and then cDNA was synthesized from each dilution (as described above).

PCR reactions were carried out in duplicates using SYBRGreen I as a fluorescent detection dye and they were performed in the real-time PCR system Mx 3000P (Stratagene, La Jolla, CA). Background-subtracted fluorescences were used for data analysis by all tested methods except for the sigmoid curve-fitting method, where raw fluorescences were used (see below). Specific oligonucleotide primers for amplification of mouse interleukin 1 beta (IL-1β), interleukin 6 (IL-6), tumor necrosis factor alpha (TNF-α), granulocyte macrophage-macrophage colony stimulating factor (GM-CSF), beta actin (ACTB), hypoxanthine guanine phosphoribosyl transferase 1 (HPRT) and succinate dehydrogenase complex subunit A (SDHA) were used (for sequences of the primers and PCR reaction conditions see [25]).

Amplification of luciferase mRNA: 1 μl of each cDNA (six cDNAs was prepared, see above) was amplified in 25 μl PCR containing 1× SYBR Green/ROX PCR Master Mix (PA-012, SuperArray Bioscience Corp., Frederick, MD), and 0.4 μM primers; 40 amplification cycles at 95°C for 20 s, 60°C for 60 s, and 82°C for 20 s (fluorescence acquiring) was used. Primers specific for the luciferase sequence (5'GCTTACTGGGACGAAGACGAAC3', 5'CTTGACTGGCGACGTAATCCAC3') amplified a 247 bp PCR product.

### Normalization of cytokine mRNAs quantity

To ensure correctness of the quantification we normalized cytokine (IL-1β, IL-6 TNF-α, and GM-CSF) expression to the three reference genes (ACTB, SDHA, and HPRT) whose expression was found to be stable in our previous work [25]. After determining quantities of mRNAs (*R*_0 _values) of the three reference genes in each sample (using tested methods of real-time PCR data analysis) the sample normalization factor was calculated as a geometric mean of the three *R*_0 _values. GeNorm software was utilized for the calculation [13]. The amount of cytokine mRNA in each sample (cytokine *R*_0 _value) was then divided by the normalization factor of the sample.

### Data analysis

The mathematical equations used in most methods for analyzing data obtained from real-time PCR are derived from the basic formula describing the PCR amplification in the exponential phase of the reaction:

*X*_*n *_= *X*_0 _× (*E *+ 1)^*n*^

where *X*_*n *_is the amount of PCR product at cycle *n*, *X*_0 _is the starting amount of PCR template (which we are interested in) and *E *is the amplification efficiency which can have a value between 0 (no amplification) and 1 (doubling of the PCR product in each amplification cycle). In fluorescent real-time PCR it is assumed that accumulation of reporter dye fluorescence (R, fluorescence readings after background subtraction) is proportional to the accumulation of PCR amplification product, and equation 1 can then be written as:

*R*_*n *_= *R*_0 _× (*E *+ 1)^*n*^

and starting fluorescence *R*_0 _can be then calculated as:

*R*_0 _= *R*_*n*_/(*E *+ 1)^*n*^

where *R*_*n *_is the intensity of reporter dye fluorescence (proportional to the amount of PCR product) at cycle *n*, and R_0 _is the theoretical starting fluorescence which is proportional to the amount of starting PCR template. Thus, the *R*_0 _value represents the target quantity expressed in arbitrary fluorescence units. There are several techniques based on this equation which are used for calculating the *R*_0 _value. In threshold-based techniques where "Ct" (the number of amplification cycles needed to reach the fluorescence threshold) is measured, "*n*" in Eq 3 can be replaced by "*Ct*":

*R*_0 _= *R*_*Ct*_/(*E *+ 1)^*Ct*^

"*R*_*Ct*_" then represents the threshold fluorescence which can be set for each of the compared amplifications individually, or a threshold value (*R*_*Ct*_) common for all compared amplifications can be used. In the latter case, the numerical value of *R*_*Ct *_in Eq 4 can be ignored (replaced for example by 1.0) and the *R*_0 _value can be calculated:

*R*_0 _= 1/(*E *+ 1)^*Ct *^or *R*_0 _= (*E *+ 1)^-*Ct*^

Other possibilities to obtain the *R*_0 _and *E *values are techniques which use relative standard curve, linear regression (utilizing equations derived from the basic formula – Eq 1) or fitting the PCR process to the sigmoid function (see below). The quantities determined from the relative standard curve as well as the quantities determined with the other methods were designated as „R_0_“ throughout the manuscript.

### Relative standard curve method

For each gene, standard cDNAs (see above) were amplified along with sample cDNAs in the same PCR run. Standard curves were generated by Mx 3000P 2.0 software (Stratagene). The threshold fluorescence common for all compared samples was set into the exponential phase of the amplifications by the Mx 3000P system. The target mRNA quantity in each sample (*R*_0_) was determined from the relative standard curve (using sample Ct values) and expressed in arbitrary units corresponding to the dilution factors of the standard RNA preparation. Amplification efficiency (*E*) representative for each gene was determined using equation of the standard curve:

*Ct *= -1/*log *(*E *+ 1) × log Ro + log R/log (E + 1)

*E *= 10^-1/*Slope *^- 1;   *slope *= -1/log [E + 1]

### Comparative Ct method

Amplification efficiency (*E*) for each gene was determined from the relative standard curve (see above). The Ct value for each reaction was determined by the real-time PCR system Mx3000P setting the threshold fluorescence (common for all compared samples) into the exponential phase of the amplifications). The target mRNA quantity in each sample (*R*_0_) was then calculated from equation 5.

### LinRegPCR method

A computer program entitled LinRegPCR developed by Ramakers *et al.*[7] utilizes linear regression analysis of fluorescence data from the exponential phase of PCR amplification to determine the target mRNA quantity (*R*_0_) as well as the amplification efficiency (*E*). Following equations were used:

*log R *= *log *(*E *+ 1) × *n *+ *log R*_0_;   *intercept *= *log R*_0_, *slope *= *log *[*E *+ 1]

*R*_0 _= 10^*Intercept*^

*E *= 10^*Slope*^- 1

LinRegPCR software utilizes an iterative algorithm (considering the number of data points, regression coefficient and slope of the regression line) for the selection of the exponential phase in each PCR amplification. We analyzed our fluorescence data using this software and obtained the *R*_0 _and *E *values for each reaction. Since ANOVA detected no significant differences in amplification efficiencies between the sample groups (Sh, Un, A, B, C) we also applied a combined analysis using an average *E *value (arithmetical mean of *E *values of all samples). In the combined analysis ("LinRegPCR-Ct method"), the *R*_0 _was calculated from equation 4 using the average *E *value and threshold fluorescence values (R) with corresponding Ct values (determined by the real-time PCR system Mx3000P).

### DART-PCR method

DART-PCR (Data Analysis for Real-Time PCR) Excel workbook developed by Peirson *et al.*[8] determines the *E *value of each individual reaction using linear regression analysis of the fluorescence data from the exponential phase of each amplification (Eq 10). For the selection of the exponential phase a midpoint (M) for each PCR amplification is calculated, using maximal and minimal fluorescence levels. DART-PCR determines the Ct value for each reaction, offering the possibility of using individual threshold fluorescences or a threshold fluorescence common for all compared reactions (the second possibility was used in this study). Target mRNA quantity (*R*_0_) is then determined using equation 4. One-way analysis of variance (included as a component of DART-PCR workbook) detected no significant differences in amplification efficiencies between the sample groups (Sh, Un, A, B, C), so we also used the average *E *value (arithmetical mean of *E *values of all samples) for the *R*_0 _calculation in each sample. Since the DART-PCR requires inputting of fluorescence data in triplicates and our PCR reactions were carried out in duplicates, we created the "third replicate" as the mean value of our duplicate fluorescence readings.

### Liu and Saint method using exponential model of PCR ("Liu & Saint-exp method")

Two arbitrary fluorescence thresholds (lower *R*_1 _and higher *R*_2_, common for all compared samples) were manually set in the exponential phase of amplification curves and corresponding Ct values (provided by the Mx 3000P) were recorded. For the thresholds selection the semilogarithmic graph of the amplification curves (log of fluorescences – log *R*, against cycle number – *n*) was used because the exponential phase of PCR amplification can be simply identified on the graph (fluorescent data acquired in the exponential phase of the reaction produce a straight line on the semilogarithmic graph). Amplification efficiency (*E*) of each reaction was calculated using equation:

*E = (R*_2_/*R*_1_)^1/(*Ct*2-*Ct*1) ^- 1

The target mRNA quantity (*R*_0_) was calculated using equation 4. One-way analysis of variance (ANOVA) detected no significant differences in amplification efficiencies between the sample groups (Sh, Un, A, B, C), so the average *E *value (arithmetical mean of *E *values of all samples) was also used for the *R*_0 _calculation in each sample (using equation 4).

### Sigmoid curve-fitting (SCF) method

Raw fluorescences (i.e. fluorescences without background subtraction) from Mx 3000P were fitted to the four-parametric sigmoid function using the nonlinear regression function of SigmaPlot (Version 10, Systat Software, Richmond, CA, USA). Following equations were used:

*R*_*na *_= *R*_*b *_+ *R*_*n *_= *R*_*b *_+ *R*_*max*_/1 + *e*^-((*n*-*n*1/2)/*k*)^

*R*_0 _= *R*_*max*_/1 + *e*^(*n*1/2/*k*)^

where *R*_*na *_is the aggregate reaction fluorescence at cycle *n*, *R*_*b *_is the background reaction fluorescence, *R*_*n *_is the fluorescence generated by the PCR product at cycle *n*, *R*_*max *_is the maximal fluorescence generated by the PCR product, *n*_1/2 _is the cycle number at which fluorescence reaches half of the *R*_*max*_, *k *describes the slope of the sigmoid curve.

Repetitive regression analyses with sequential removal of the last amplification cycle (the cut-off cycle) were performed until the curve fitting failed, due to insufficient data. The *R*_0 _value was calculated at each curve-fitting using equation 13. For all amplification curves, the graph of dependence of the calculated *R*_0 _values on the cut-off-cycle was constructed and the minimum *R*_0 _value was selected as the resulting *R*_0 _value – the target mRNA relative quantity [11]. For the data treatment a macro for SigmaPlot provided by Qiu *et al.*[24] was utilized. In some instances the cut-off cycle with the minimal *R*_0 _value was located in the linear region of the amplification curve. The resulting *R*_0 _value was then selected manually by shifting the cut-off cycle into the region located between end of the linear amplification and entry of the reaction into the plateau phase (i.e. into "upper arc" of the amplification curve).

### Intra-assay and inter-assay variability

In the intra-assay variability experiment, fifteen replicate PCR reactions amplifying IL-1β or IL-6 were ran. In the inter-assay variability experiment, eight PCR reactions amplifying IL-1β or IL-6 were ran in eight separate PCR (Mx 3000P) runs. The fluorescent data obtained from each reaction replicate were then transformed into mRNA quantity (the *R*_0 _value) by the tested methods for real-time PCR data analysis. Arithmetical means, standard deviations (SD) and coefficients of variation (CV) were then calculated from the determined *R*_0 _values (15 and 8 replicates for each gene, respectively).

### Statistics

All statistics were performed using Statistica (StatSoft, Tulsa, OK). To enable better comparison of the results obtained from the different methods of real-time PCR data analysis the values of target mRNA quantities (*R*_0_) were transformed percentually (setting the median of *R*_0 _values of all samples as 100%). Differences in amplification efficiencies between the sample groups were assessed using one-way analysis of variance (ANOVA). The Kruskal-Wallis test was used for the comparison of differences in normalized cytokine expression between groups, and the Mann-Whitney U test was used to compare differences between the group of untreated colitic animals (Un) and other groups of animals. Values of P < 0.05 were considered as significant.

## Authors' contributions

SC conceived and designed the study, analyzed and interpreted the data, and drafted the manuscript. AB participated in the fluorescent data analysis and interpretation, and in the manuscript preparation. JK participated in the data interpretation and manuscript preparation. All authors read and approved the final manuscript.
